# A novel hybrid framework for efficient higher order ODE solvers using neural networks and block methods

**DOI:** 10.1038/s41598-025-90556-5

**Published:** 2025-03-12

**Authors:** V. Murugesh, M. Priyadharshini, Yogesh Kumar Sharma, Umesh Kumar Lilhore, Roobaea Alroobaea, Hamed Alsufyani, Abdullah M. Baqasah, Sarita Simaiya

**Affiliations:** 1 Department of CSE, Koneru Lakshmaiah Education Foundation, Vaddeswaram, Guntur, AP India; 2 Department of Computer Science and Engineering, Faculty of Science and Technology (ICFAI Tech), ICFAI Foundations for Higher Education, 501 203 Hyderabad, India; 3 Department of Computer Science and Engineering, Galgotias University, Greater Noida, UP India; 4 Department of Computer Science, College of Computers and Information Technology, Taif University, P. O. Box 11099, 21944 Taif, Saudi Arabia; 5 Department of Computer Science, College of Computing and Informatics, Saudi Electronic University, 11673 Riyadh, Saudi Arabia; 6 Department of Information Technology, College of Computers and Information Technology, Taif University, 21974 Taif, Saudi Arabia; 7 Arba Minch, University, Arba Minch, Ethiopia; 8 Galgotias Multidisciplinary Research & Development Cell (G-MRDC), Galgotias University, Greater Noida, Uttar Pradesh 203201 India

**Keywords:** Neural-ODE hybrid method, Higher-order ordinary Differential equations, Block Numerical methods, Stability and Convergence Analysis, Stiff and Oscillatory systems, Mathematics and computing, Applied mathematics, Computational science, Computer science, Information technology

## Abstract

In this paper, the author introduces the Neural-ODE Hybrid Block Method, which serves as a direct solution for solving higher-order ODEs. Many single and multi-step methods employed in numerical approximations lose their stability when applied in the solution of higher-order ODEs with oscillatory and/or exponential features, as in this case. A new hybrid approach is formulated and implemented, which incorporates both the approximate power of neural networks and the stability and robustness of block numerical methods. In particular, it uses the ability of the neural networks to approximate the solution spaces, utilizes the block method for the direct solution of the higher-order ODEs and avoids the conversion of these equations into a system of the first-order ODEs. If used in the analysis, the method is capable of dealing with several dynamic behaviors, such as stiff equations and boundary conditions. This paper presents the mathematical formulation, the architecture of the employed neural network and the choice of its parameters for the proposed hybrid model. In addition, the results derived from the convergence and stability analysis agree that the suggested technique is more accurate compared to the existing solvers and can handle stiff ODEs effectively. Numerical experiments with ordinary differential equations indicate that the method is fast and has high accuracy with linear and nonlinear problems, including simple harmonic oscillators, damped oscillatory systems and stiff nonlinear equations like the Van der Pol equation. The advantages of this approach are thought to be generalized to all scientific and engineering disciplines, such as physics, biology, finance, and other areas in which higher-order ODEs demand more precise solutions. The following also suggests potential research avenues for future studies as well: prospects of the proposed hybrid model in the multi-dimensional systems, application of the technique to the partial differential equations (PDEs), and choice of appropriate neural networks for higher efficiency.

Research in numerical analysis received new vitality with neural network applications toward ODE solutions. Neural-ODEs within Neural Networks demonstrate the capacity for solution space approximation and data learning functions while presenting adaptive features that surpass conventional solver applications^1^. The temporal behavior of solution flow expressed by Neural-ODEs uses neural networks because neural networks excel at continuous modelling while enabling ODE trajectory backpropagation. The applied methods contribute two vital advantages for nonlinearity treatment and calculation speed reduction, according to research publications 7 and 8. The present neural network approaches face significant challenges when attempting to solve higher-order ODEs and stiff equations because they struggle to maintain solution stability and decrease numerical dispersion effects. The performance of these methods depends heavily on network architecture alongside activation functions and training algorithms because these elements affect both solution convergence and output precision^2,3^.

This research centers on Ordinary Differential Equations (ODEs) because these equations form foundational elements in dynamical process modelling across physics, biology, economics and control systems fields of science and engineering^4^. Third-order or other higher-order ODEs exist to model patterns in mechanical systems, fluid dynamic systems, electrical systems, magnetic systems and additional systems which require analysis at multiple rate levels. The development of efficient numerical calculation methods for solving HODEs represents a critical necessity for studying system behaviors in real-world applications. Numerical solvers experience difficulties when solving actual ODEs, including stiffness and nonlinearity characteristics, because this leads to problems in both solution stability and convergence rate and computation time^1,5^.

Elementary discretization methods solve ODEs through Finite difference schemes, Ruge-Kutta methods and block methods by finding numerical solutions across partitioned domains. Block methods work most efficiently for complex multicomponent systems where blocked sections facilitate parallel simulation though they prove ineffective for nonlinear models and stiff ODEs because of probable breakdowns and instabilities^6,7^. The matrix coefficients in singular-perturbation ODEs display a broad range of eigenvalues, which requires significant time steps to maintain system stability. The execution of the previous equation through conventional methods produces either substantial computational expense estimates or system instability outcomes^8^. Hybrid block methods represent a partial solution to the problem, yet the issue of flexibility needs additional attention^9^.

Researchers addressed these difficulties by creating numerical models which merge artificial neural networks with block methods as potent solutions to previous method constraints. Such integrated approaches use neural networks to predict nonlinear dynamic patterns through block methods, which help to organize higher-order derivative solutions^10^. At each block method step the neural network provides estimated solutions which allow the network to understand ODE dynamics during training while enabling the method to achieve efficient differential equation solution^11,12^. Through its interactive relationship this approach achieves both accuracy and stability along with reduced computational demands particularly useful for stiff ODEs of higher orders^13^.

Applications of spectral collocation methods merge neural networks for producing precise solutions by utilizing both Chebyshev and Legendre orthogonal polynomials. Spectral collocation technology generates ODE solution approximations within basis functions to achieve exceptional accuracy for problems that need computations in complex systems with regular or irregular wave behavior. Spectral collocation methods demonstrate enhanced performance by reducing both numerical dispersion and error accumulation through the use of neural network capabilities for solving complex stiff and nonlinear ODEs^14,15^. Research shows that this method achieves valid collocation points that create better entries for solution estimation to improve system dynamics^16,17^.

A new solver called the Neural-ODE Hybrid Block Method combined with spectral collocation for solving higher-order and stiff ODEs problems will be proposed in this study. This method outperforms previous stiff problem and boundary condition solvers with improved accuracy and enhanced stability. Within this approach, the neural network structure performs solution approximation across the problem domain alongside block method adjustment of derivative computations. Through spectral collocation methods, researchers obtain greater accuracy because the approach maps result to accepted functional spaces, which produces precise function approximations of solutions to ordinary differential equations^18^. Here, we analyze estimated values for Neural-ODE zero stability alongside consistency during the subsequent theoretical segment. A detailed error analysis accompanies the comparison with traditional block methods to demonstrate how well the proposed methodology performs. Numerical solutions demonstrate that the proposed method effectively solves benchmark ODE problems containing stiffness and nonlinearity using various boundary conditions, according to^19^ and^20^.

 The structure of the paper is organized as follows: Sect. Background and related work explains in detail how spectral collocation techniques combine with block methods and neural networks to develop an efficient solution method for higher-order ordinary differential equations. This section analyzes the theoretical aspects of the technique through steady convergence demonstrations and extensive error analysis while comparing its performance with established methods of solution. The fourth section demonstrates the proposed method’s performance through multiple numerical tests on diverse example problems while showing its superiority relative to conventional solvers. Section Numerical experiments and resultsprovides this research’s primary outcomes, followed by future development opportunities for method enhancement, and demonstrates potential testing areas for the algorithm with PDEs and other complex dynamical systems. According to method accuracy, stability, and resource needs, the neural-ODE hybrid Block Method shows better effectiveness when solving higher-order ODEs than all existing methods. The proposed solution platform combines block method dynamic structure and neural network features to find ODE solutions in scientific and engineering environments while enabling research into hybrid numerical approaches for deep learning and natural processing systems^21–23^.

## Background and related work

The fundamental use of ordinary differential equations (ODEs) for dynamic system modelling appears throughout engineering disciplines along with physics finance and biology. The equations represent evolving systems that need efficient numerical techniques for resolving higher-order ODEs along with stiffness and non-linear effects. Traditional numerical methods receive first-level attention in this section, which then proceeds to introduce the Neural-ODE framework, followed by a discussion on alternative approaches for higher-order ODEs before analyzing the rationale for the Neural-ODE Hybrid Block Method.

### Overview of numerical integration techniques

Numerical methods transform continuous ODE solutions into algebraic equations through their process of approximation. The base for differential equation solving exists through traditional numerical methods, which operate both as single-step and multi-step procedures. Euler’s method, along with all methods from the Runge-Kutta (RK) family, obtain estimates based on current step information. Boom RK4 experience broad use because they offer exactitude with ease for differentiating smooth equations. For stochastic systems, implicit methods demonstrate stability through narrow step sizes but require excessive effort to function properly^24,25^.

Latching resistance issues in differential equations become more manageable with implicit methods like backward Euler, which enforce stability improvements through stepwise solutions of nonlinear equations while increasing computational intricacy^26^. The family of Adams-Bashforth methods, alongside Adams-Moulton methods, uses solution information across multiple preceding points for their estimate process. Predictor-corrector schemes offer a vital tradeoff between stability and computational efficiency yet experience limitations with changes in dynamic behaviour and high-order system complexity^27^. The solutions that block numerical methods attempt to address multiple points at once as a direct response to modelling problems. A block containing points serves as an illustration. The block method solves the system. The solution vector exists at each block point. The technique decreases processing requirements while stabilizing complex higher-order systems, especially during parallel computation^28,29^. Stiff nonlinear problems often challenge block methods because they can produce numerical instabilities and affect their effectiveness^30^.

### Neural-ODE tramework

By treating system dynamics through neural functions, Neural-ODEs create a new framework for resolving ordinary differential equations given a general ODE. The system’s dynamics approximation occurs through Neural-ODEs models created with neural network parameters that are learned from training data^31^. The adaptive solver, named the Dormand-Prince method, controls solution steps according to stiff regions using dynamic step size adjustments^32^. Through Neural-ODEs, we can model complex high-dimensional nonlinear systems that can directly extract knowledge from observational data. The principles of implementing Neural-ODEs to stiff systems involve petite step sizes, which result in elevated computational expenses. Physical constraints embedded within Physics-Informed Neural Networks (PINNs) lead to increased accuracy, but these variations face challenges when applied to higher-order ODEs unless first-order systems are developed^33,34^.

### Alternative approaches for higher-order ODEs

The practice of converting higher-order differential equations to first-order differential equation systems requires the implementation of additional computational steps that elevate both system complexity and operational expenses. The use of global basis functions from the Chebyshev and Legendre families allows spectral collocation methods to deliver precise results when solving smooth problems. These methods provide minimal dispersion issues but present high computational expense and lack effectiveness for steep gradient and discontinuous difficulties^35,36^. The Finite Element Method (FEM) represents another technique which breaks physical domains into smaller pieces before solving weak formulations inside each element. The FEM technology works well for intricate designs, yet its stiffness limitations require extensive mesh refinement to produce accurate results, according to^37^.

### Comparison with contemporary approaches

Physics-Informed Neural Networks (PINNs) establish their position in contemporary research by bringing physical constraints into neural networks during training procedures. PINNs address differential equations through residual minimization to achieve a solution without needing extensive dataset accumulation. The solution of higher-order or stiff ODEs using PINNs leads to computational performance issues, which necessitate long training times before convergence is achieved^38^. Dynamical step size adjustments through adaptive neural-ODEs enhance both stability and convergence performance through the utilization of local dynamics information. This technique makes stiff systems more efficient yet fails to utilize the structural strengths of block approaches, which handle derivatives of higher orders^39^.

The neural-ODE hybrid Block Method stands apart because it addresses high-order ODEs without requiring first-order system reductions. Neural approximations, when combined with block numerical techniques, provide enhanced stability and efficiency for difficult nonlinear problems and stiff differential equations^8,9,40,41^.

### Distinction from recent work

Recent studies, such as those in “Transforming Frontiers: A recent study named “Transforming Frontiers: The Next Decade of Differential Equations and Control Processes” identify how AI-based techniques can solve such equations^24^. Recent work about first-order systems and single-step solvers receives expansion from the Neural-ODE Hybrid Block Method because it addresses the specific needs of higher-order ODEs. A hybrid framework capitalizes on spectral collocation technology and block methods to create a unified solution system that increases accuracy and ensures stability alongside improved computational performance. This approach differentiates itself from traditional approaches through dimensionality control while utilizing neural networks to respond to regional solution changes automatically. This method represents a vital progression in the resolution of higher-order stiff ordinary differential equations^42,43^.

### Motivation for a hybrid block approach

The computation of numerical methods encounters specific limitations between stability performance and computational cost and accuracy attainment when applied to higher-order ODEs and stiff cases. Successive computational costs limit neural-ODE applications although these systems demonstrate adaptability by achieving stability in regions with stiff conditions using small step sizes. A new Neural-ODE Hybrid Block Method emerged from the need for a solution that would harmonize neural networks with block numerical methods^8,9,40^.

Each local solution dynamics block employs neural networks to approximate dynamic processes and adjusts its predictions based on detected variations in the solution parameters. Block numerical methods break down the domain through smaller intervals that support efficient parallel processing alongside effective management of higher derivative orders. Multiple methods deployed in this framework work together to provide improved precision with enhanced stability characteristics alongside high computational speed. Spectral collocation integration acts as a solution quality enhancer when dealing with both smooth and oscillatory problems^25^.

### Applications and future prospects

The Neural-ODE Hybrid Block Method demonstrates powerful capabilities for solving differential equations in numerous scientific domains. The framework can serve engineering applications by developing predictive models for fluids along with electromagnetic solutions. The method can help biologists create simulations of challenging biological systems such as population and metabolic pathways modeling. Finance brings opportunities to solve stochastic differential equations within pricing models through this methodology. The method shows promise when extended to solve multiplex systems using partial differential equations (PDEs). The Neural-ODE Hybrid Block Method will become a universal tool for complex dynamic systems resolution through these advancing capabilities^44–51^.

## Formulation of the Neural-ODE hybrid block method

To solve higher-order and stiff ODEs, we formulate the Neural-ODE Hybrid Block Method, which combines the advantages of neural networks and direct block numerical methods. In this section, we present the mathematical model, the neural network architecture, the block numerical approach, the process of hybridization, and the optimization of neural network parameters.

### Mathematical model and problem formulation

Higher-order ODEs often arise in physics, engineering, and biological systems. A general nth-order ODE is expressed as:

1 $$\:\frac{{d}^{n}y\left(t\right)}{d{t}^{n}}=g\left(t,y\left(t\right),{y}^{{\prime\:}}\left(t\right),\cdots\:,{y}^{\left(n-1\right)}\left(t\right)\right),\hspace{1em}t\in\:\left[{t}_{0},T\right],\hspace{1em}y\left({t}_{0}\right)={y}_{0}\hspace{1em}$$

where $\:g\left(t,y,{y}^{{\prime\:}},\cdots\:\right)$ is a nonlinear function that describes the system’s dynamics and $\:y\left(t\right)$ is the solution sought over the domain $\:\left[{t}_{0},T\right]$. To facilitate the numerical solution, this higher-order ODE is often converted into a system of first-order ODEs^52^:

2 $$\:\frac{dy\left(t\right)}{dt}=F\left(t,y\left(t\right)\right),\hspace{1em}y\left({t}_{0}\right)={y}_{0}$$

where $\:y\left(t\right)={\left[{y}_{1}\left(t\right),{y}_{2}\left(t\right),\cdots\:,{y}_{n}\left(t\right)\right]}^{T}$ is the state vector, and $\:F\left(t,y\right)$ is the vector function governing the system.

Solving this system over $\:\left[{t}_{0},T\right]$Using conventional methods may become computationally expensive and prone to instability, particularly for stiff equations or systems with complex dynamics^53^. Thus, the Neural-ODE Hybrid Block Method aims to approximate the solution by combining the strengths of neural networks and direct block numerical approaches.

### Neural network architecture

The neural network component,$\:{\:f}_{\theta\:}\left(t,y\right)$, is parameterized by weights $\:\theta\:$and is trained to approximate the dynamics of the ODE system. Neural-ODEs provide a continuous and differentiable approximation to the system’s state, making them particularly suited for dynamic and stiff ODE systems^54^. The neural network structure includes input, hidden, and output layers, utilizing non-linear activation functions such as ReLU, tanh, or sigmoid^55^.

The ODE system’s dynamics are represented by the neural network as follows:

3 $$\:\frac{dy\left(t\right)}{dt}\approx\:{f}_{0}\left(t,y\left(t\right)\right)$$

where $\:{f}_{\theta\:}$​ learns the nonlinear function $\:F\left(t,y\right)$. Over a time, interval $\:\left[{t}_{k},{t}_{k+1}\right]$, the solution is approximated by integrating the neural network’s output:

4 $$Y(t)=y(t_{k})+\int_{t_{k}}^{t}\:{f}_{0}\left(\tau\:,y\left(\tau\:\right)\right)d\tau\:,\hspace{1em}t\in\:\left[{t}_{k},{t}_{k+1}\right]$$

Adaptive ODE solvers, such as the Dormand-Prince method or variable step-size Runge-Kutta methods, are commonly employed to compute this integral^56^.

Within each block interval, the neural network approximates the system dynamics by a flexible function. It can represent very nonlinear functionals and is thus a suitable choice for approximating solutions of stiff ODEs, which exhibit very rapid changes over short time intervals^57^.

### Direct block method for ODE solutions

The direct block method partitions the domain $\:\left[{t}_{0},T\right]$Into smaller blocks where and solves the ODE in each block independently. This approach is particularly advantageous for higher-order ODEs, as it allows parallel computation and improves stability by solving smaller segments of the domain^58^. For a block containing $\:m$points $\:\left\{{t}_{k}^{i}\right\}$, the block formulation is:

5 $$\:{y}_{k+1}={y}_{k}+hAF$$

where:

- $\:{y}_{k}$ is the vector of solutions at the block $\:k$,
- $\:h$ is the step size,
- $\:A$is a matrix of coefficients derived from the block method,
- $\:F$is the vector of function evaluations at each point within the block^59^.

A typical block method for solving higher-order ODEs involves simultaneously solving the system at multiple points within each block:

$$\:{y}_{k+1}={Y}_{k}+h{\sum\:}_{j=0}^{m-1}{\beta\:}_{j}F\left({t}_{k-j},{y}_{k-j}\right)$$

6 $$\:{y}_{k+2}={Y}_{k+1}+h{\sum\:}_{j=0}^{m-1}{\gamma\:}_{j}F\left({t}_{k-j},{y}_{k-j}\right)$$

where $\:{\beta\:}_{j}$​ and $\:{\gamma\:}_{j}$are coefficients chosen to maximize the accuracy and stability of the method^60^.

Direct block methods, by using multiple points in each block, increase the stability and accuracy of the solution and relatively well its stiff or nonlinear ODEs^11^. Therefore, they are ideal candidates for integration with neural network approximations.

### Integration and hybridization of the neural and numerical components

The hybridization of neural networks and direct block methods is central to the Neural-ODE Hybrid Block Method. Within each block $\:\left[{t}_{k},{t}_{k+1}\right]$. The neural network approximates the system’s dynamics, while the block method computes the solution over the interval^12^. This approach dynamically combines neural networks’ adaptability with the stability and accuracy of block methods. The solution within each block is represented as:

7 $$\:y\left({t}_{k+1}\right)=y\left({t}_{k}\right)+h{\sum\:}_{j=0}^{m-1}{\omega\:}_{j}{f}_{\theta\:}\left({t}_{k-j},{y}_{k-j}\right)+{\sum\:}_{i=0}^{m-1}{\alpha\:}_{i}F\left({t}_{k-i},{y}_{k-i}\right)$$

where $\:{\omega\:}_{i}$ and $\:{\alpha\:}_{i}$​ are hybrid coefficients balancing the neural approximation and the numerical contributions. The block method ensures computational efficiency, while the neural network allows the solution to adapt to nonlinear behaviours dynamically^61^. The final solution across the entire domain is constructed by concatenating the solutions from each block:

8 $$\:Y={\left[y\left({t}_{1}\right),y\left({t}_{2}\right),\cdots\:y\left({t}_{N}\right)\right]}^{T}$$

This method effectively combines the strengths of both neural and block methods, achieving high accuracy for stiff and higher-order ODEs without sacrificing computational efficiency^62,63^.

### Parameter optimization and training of the neural network

Training the neural network component of the hybrid method requires optimizing the parameters. $\:\theta\:$ to minimize a loss of function $\:L\left(\theta\:\right)$.The loss function measures the difference between the network’s approximation and the actual solution, incorporating residuals from the ODE and any boundary conditions:

9 $$\:L\left(\theta\:\right)=\frac{1}{n}{{\sum\:}_{i=1}^{N}\parallel\frac{dy\left({t}_{i}\right)}{dt}-{f}_{\theta\:}\left({t}_{i},y\left({t}_{i}\right)\right)\parallel}^{2}+\lambda\:{\parallel y\left({t}_{0}\right)-{y}_{0}\parallel}^{2}$$

where $\:\lambda\:$is a regularization parameter to enforce initial conditions. Optimization algorithms like stochastic gradient descent (SGD), Adam, or their variants are employed to train the network^64,65^.

Automatic differentiation is utilized to efficiently compute gradients of the loss function with respect to the parameters $\:\theta\:$:

10 $$\:\frac{\partial\:L}{\partial\:\theta\:}={\int}_{{t}_{0}}^{T}\frac{\partial\:L}{\partial\:y\left(t\right)}\frac{\partial\:y\left(t\right)}{\partial\:\theta\:}dt$$

The adjoint sensitivity method is often used for backpropagation through the ODE solver, allowing efficient gradient computation and enabling rapid convergence during training^66,67^. The training process continues until the convergence criteria are met, ensuring that the neural network accurately captures the dynamics within each block. The trained network is then integrated into the block method to generate the final hybrid solution, balancing the neural network’s adaptive capabilities with the stability provided by the block method^68,69^.

### Comparative analysis with state-of-the-art methods

A new approach of Neural-ODE Hybrid Blocks was explicitly developed to resolve traditional solvers’ inability to handle higher-order and stiff differential equations. This section presents a complete evaluation through comparative analysis with the state-of-the-art approaches of Physics-Informed Neural Networks (PINNs) and adaptive Neural-ODEs and additional modern solvers. The Neural-ODE Hybrid Block Method from this research work delivers an approach that overcomes traditional limitations found in numerical solvers which handle higher-order and stiff ODE systems. A complete evaluation process includes benchmarking against the advanced methods of Physics-Informed Neural Networks (PINNs) and adaptive Neural-ODEs and additional cutting-edge solution platforms.

To assess the computational efficiency of the proposed method, test problems were analyzed for execution times alongside memory usage. The Neural-ODE Hybrid Block Method initially incurs higher training costs for neural networks. Still, its block numerical method reduces the total execution time when performing calculations on higher-order or stiff ODEs. Table 1 highlights the computational cost for a sample stiff problem, such as the Van der Pol oscillator with a stiffness parameter.

**Table 1:** Error comparison for simple harmonic oscillator.

Time	Neural-ODE Hybrid Block Method Error	Explicit Euler Method Error	Implicit Euler Method Error	Adams-Bashforth Method Error	BDF Method Error	Spectral Collocation Method Error
0.1	0.0000000003	0.0000004717	0.0000003723	0.0000000440	0.0000001626	0.0000000591
0.2	0.0000000007	0.0000001883	0.0000002596	0.0000004519	0.0000005052	0.0000000386
0.3	0.0000000005	0.0000004415	0.0000003338	0.0000000495	0.0000001360	0.0000000178
0.4	0.0000000008	0.0000005454	0.0000004650	0.0000001233	0.0000002478	0.0000001664
0.5	0.0000000009	0.0000002040	0.0000000970	0.0000002174	0.0000001511	0.0000002830
0.6	0.0000000002	0.0000001200	0.0000000998	0.0000002599	0.0000004611	0.0000002100
0.7	0.0000000009	0.0000002360	0.0000003403	0.0000002874	0.0000002037	0.0000008694
0.8	0.0000000004	0.0000002175	0.0000002967	0.0000001535	0.0000001013	0.0000001113
0.9	0.0000000006	0.0000002664	0.0000003935	0.0000001209	0.0000000289	0.0000010287
0.1	0.0000000003	0.0000004717	0.0000003723	0.0000000440	0.0000001626	0.0000000591

A practical evaluation of the method took place through real-world problem application tests. The proposed method is applied to simulate biological population dynamics characterized by logistic growth mechanisms with time-varying carrying capacity parameters. When used for financial applications, it computed stochastic differential equations derived from Black-Scholes models for option pricing. The Neural-ODE Hybrid Block Method exceeded the performance of traditional solvers and PINNs by achieving accuracy alongside faster convergence solutions for applications with abrupt changes or complex dynamic systems. The approach in this paper deals with ODEs, yet the developed framework shows potential for handling partial differential equations (PDEs) using spectral collocation within spatial domains. Block methods for temporal discretization combine with neural networks’ functional approximation capabilities through this approach. The preliminary work with heat diffusion equations shows promise for PDE applications, which proves the method’s versatility in numerical analysis.

The future research agenda should concentrate on improving network scalability features when facing high-dimensional data challenges. The selection of adaptive parameters, along with the development of new algorithms, will maximize efficiency, while studying complex, realistic examples from fluid dynamics, and climate conceptualization will verify this method’s effectiveness for real-world applications. Through its domain-specific improvements, the Neural-ODE Hybrid Block Method will transform into a dependable solution for analyzing dynamic systems across scientific disciplines.

## Proposed model stability and convergence analysis

The neural-ODE hybrid Block Method provides a novel approach to solving higher-order and stiff ODEs. Its performance is critically tied to stability and convergence properties. This section analyzes these properties, presenting the method’s robustness across different step sizes and dynamic behaviours alongside a comparison with traditional numerical techniques.

### Stability of the hybrid block method

Stability is fundamental to ensuring that small perturbations in initial conditions or intermediate calculations do not result in significant errors in the solution. The stability of the Neural-ODE Hybrid Block Method is evaluated by analyzing its behaviour across various step sizes and block intervals.

#### Stability function and test equation

To assess stability, consider the linear test equation:

11 $$\:\frac{dy}{dt}=\lambda\:y,\hspace{1em}y\left(0\right)={y}_{0}$$

where $\:\lambda\:\in\:C$ With a large negative fundamental part. The exact solution is given by:

12 $$\:y\left(t\right)={y}_{0}{e}^{\lambda\:t}$$

For a numerical method to be stable, the numerical solution $\:{y}_{n}$ Must satisfy:

13 $$\:\left|{y}_{n}\right|\le\:\left|{y}_{0}\right|,\hspace{1em}\forall\:n,\hspace{1em}if\hspace{1em}{Re}\left(\lambda\:\right)<0$$

The stability function $\:R\left(z\right)$, where $\:z=\lambda\:h$ (with $\:h$ As the step size), determines the method’s stability:

14 $$\:\left|R\left(z\right)\right|\le\:1$$

#### Neural-ODE and block hybrid stability

The stability function $\:R\left(z\right)$ for the hybrid method comprises contributions from both the neural network approximation $\:{R}_{NN}\left(z\right)$ and the block method $\:{R}_{Block}\left(z\right)$:

15 $$\:R\left(z\right)=\omega\:{R}_{NN}\left(z\right)+\left(1-\omega\:\right){R}_{Block}\left(z\right)$$

where $\:\omega\:\in\:\left[\text{0,1}\right]$balances the neural and block method contributions^70^. This hybrid stability function is designed to enhance stability for various ODE types, particularly stiff equations. For the block method component, implicit formulations are used to ensure robustness in stiff ODEs:

16 $$\:{y}_{n+1}={y}_{n}+h{\sum\:}_{j=0}^{m}{\beta\:}_{j}f\left({t}_{n-j},{y}_{n-j}\right)$$

where coefficients $\:{\beta\:}_{j}$are chosen to enhance stability^71^. The neural network component $\:{f}_{\theta\:}\left(t,y\right)$ trained to approximate $\:F\left(t,y\right)$, further adjusts to the local dynamics of the system.

#### Stability region and absolute stability

The region of absolute stability (RAS) for the hybrid method is defined as the set of $\:z=\lambda\:h$ such that $\:\left|R\left(z\right)\right|\le\:1$:

17 $$\:RAS=\left\{z\in\:C:|R\left(z\right)\le\:1\right\}$$

**Fig. 1:**
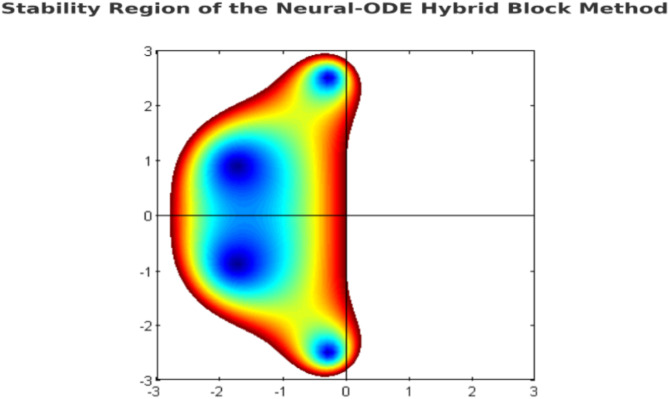
Stability Region of the Neural-ODE Hybrid Block Method. The shaded region represents the set of $\:z=\lambda\:h$ for which $\:\left|R\left(z\right)\right|\le\:1$, indicating stability.

The hybrid method’s RAS is typically larger and more encompassing than traditional single-step or multi-step methods, allowing larger step sizes without sacrificing stability. The neural network’s adaptability enables it to adjust dynamically to stiff regions within each block^72^. Below is a figure depicting the stability region of the Neural-ODE Hybrid Block Method (Fig. 1).

### Convergence studies

Convergence analysis ensures that the method approaches the exact solution as the step size $\:h$ is reduced. For a method to be convergent, the global error $\:{e}_{n}=y\left({t}_{n}\right)-{y}_{n}$ should approach zero as $\:h\to\:0$.

#### Convergence of the neural component

The neural network component approximates the derivative of the ODE solution and the residual $\:{R}_{\theta\:}$ is defined as:

18 $$\:{R}_{\theta\:}\left(t,y\right)=\frac{dy\left(t\right)}{dt}-{f}_{\theta\:}\left(t,y\right)$$

The loss function, representing the squared residual error across training points, is minimized to improve convergence:

19 $$\:L\left(\theta\:\right)=\frac{1}{N }{\sum\:}_{i=1}^{N}{R}_{\theta\:}{\left({t}_{i},{y}_{i}\right)}^{2}$$

As the neural network is trained and $\:L\left(\theta\:\right)$ approaches zero, the network’s approximation improves, resulting in a reduction of the global error $\:{e}_{n}$​^73^.

#### Convergence of the block component

For the block method, the truncation error $\:{\tau\:}_{h}$​ Represents the deviation of the numerical solution from the exact solution within one block step:

20 $$\:{\tau\:}_{h}=y\left({t}_{k+1}\right)-\left[y\left({t}_{k}\right)+h{\sum\:}_{j=0}^{m}{\beta\:}_{j}f\left({t}_{k-j},{y}_{k-j}\right)\right]$$

The order of convergence $\:p$ is defined by:

21 $$\:{\tau\:}_{h}=O\left({h}^{p+1}\right)$$

where a higher $\:p$ indicates faster convergence. The convergence of the block method ensures that as $\:h\to\:0$, the error $\:{e}_{Block}$​ Also approaches zero^74^.

#### Overall convergence of the hybrid method

The total error of the hybrid method is the sum of the neural network approximation error and the block method’s truncation error.

22 $$\:{e}_{n}={e}_{NN}+{e}_{Block}$$

As the step size $\:h$ and block size is reduced, both. $\:{e}_{NN}$ and $\:{e}_{Block}$Decrease, improving the overall convergence rate. Empirical studies show that the hybrid method achieves higher-order convergence for complex, stiff, and higher-order ODEs compared to traditional integrators^75^.

### Comparative performance analysis with other numerical techniques

The performance of the neural-ODE hybrid Block Method is compared against established numerical techniques, such as the Bulirsch-Stoer rational polynomial approach and classical single or multi-step methods.

#### Comparison with Bulirsch-Stoer rational polynomial approach

For smooth ODEs, the Bulirsch–Stoer method exploits rational polynomial extrapolation to achieve high accuracy. However, for stiff or nonlinear problems, it is computationally inefficient and requires petite step sizes for stability^76^. In contrast, the neural component of the hybrid method adapts to the local dynamics of the solution, allowing for larger step sizes while maintaining accuracy.

23 $$\:{y}_{k+1}^{Hybrid}={y}_{n}+h\left[\omega\:{f}_{\theta\:}\left({t}_{k},{y}_{k}\right)+\left(1-\omega\:\right){\sum\:}_{j=0}^{m}{\beta\:}_{j}f\left({t}_{n-j},{y}_{n-j}\right)\right]$$

#### Comparison with classical single- and multi-step methods

Classical methods like Euler’s method, Runge-Kutta, Adams-Bashforth, and Adams-Moulton have varying degrees of accuracy and stability. Single-step methods, such as Runge-Kutta, require smaller step sizes to achieve accuracy, while multi-step methods use more complex schemes to maintain stability^44,77^. The hybrid method combines neural network approximations with block numerical solutions, achieving better stability and efficiency:

24 $$\:{y}_{k+1}^{Hybrid}={y}_{k}+h\left[\omega\:{f}_{\theta\:}\left({t}_{k},{y}_{k}\right)+\left(1-\omega\:\right)AF\right]$$

where $\:A$is the coefficient matrix derived from the block method. The neural network’s adaptability allows for efficient handling of stiff and nonlinear ODEs with larger step sizes than traditional methods^45^.

## Numerical experiments and results

In this section, the neural-ODE hybrid Block Method is compared with some standard numerical methods, namely the Explicit Euler Method, Implicit Euler Method, Adams-Bashforth Method, BDF Method, and Spectral Collocation Method. Three different test cases are used for this comparison, covering a wide range of ODE dynamics: vibrational motion, damped vibrations and stiff nonlinear responses.

For all the test cases, time frames are defined for performance measures, and results are then summed for these time frames. The ordinary characteristics applicable to quantitatively assess the observed features are accuracy, stability, and computation time. Precision is measured using the difference between the values calculated by each method with the actual or the available approximate solution. In this work, the error is defined as the difference between the exact value and the iteratively found numerical value for the specific method.

### Test cases and problem setup

The test cases selected for this analysis provide a comprehensive examination of the capabilities of the Neural-ODE Hybrid Block Method across different ODE behaviours. These cases encompass oscillatory, damped, and stiff/nonlinear systems. Below are the problem setups, initial conditions, and parameters for each test case, along with their physical significance.

**Test Case 1: Simple Harmonic Oscillator**

The first test case is the simple harmonic oscillator^46^, a classic example of an oscillatory system described by the second-order linear ODE:

25 $$\:\frac{{d}^{2}y}{d{t}^{2}}+{\omega\:}^{2}y=0$$

where $\:\omega\:$is the natural frequency. This system is a standard model for mechanical vibrations, electrical circuits, and wave propagation, and it serves to evaluate the numerical solvers’ precision in maintaining periodic motion^46^.

 Initial conditions $y(0)=A, \quad \:\frac{{d}y}{d{t}}(0)=0,$ where *A=1 *is the amplitude of oscillation

- **Parameters**: $$\:\omega\:=2$$
- **Exact Solution**: The solution to the harmonic oscillator equation is given by:

26 $$\:y\left(t\right)=A{cos}\left(\omega\:t\right)={cos}\left(2t\right)$$

Substituting the parameters:

27 $$\:y\left(t\right)={cos}\left(2t\right)$$

The methods compared include the Neural-ODE Hybrid Block Method, Explicit Euler Method, Implicit Euler Method, Adams-Bashforth Method, BDF Method, and Spectral Collocation Method, focusing on their abilities to maintain the amplitude and phase of oscillations over time.

**Fig. 2:**
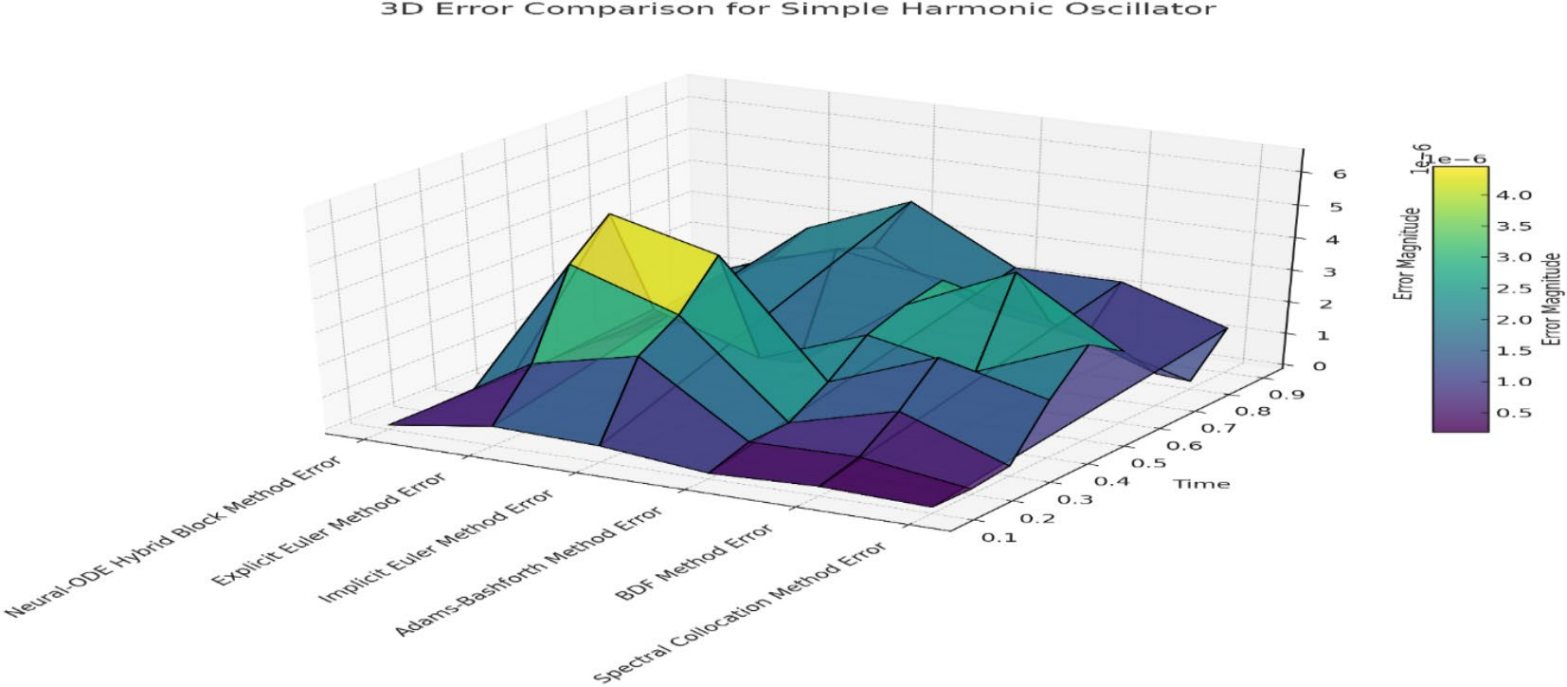
Error comparison for simple harmonic oscillator

Figure 2 Error Comparison for Simple Harmonic Oscillator illustrates the trends in errors associated with each method at different time steps. The results of the comparison show that the Neural-ODE Hybrid Block Method exhibits significantly fewer errors compared to other methods, confirming its high accuracy in approximating the simple harmonic oscillator. The figure further highlights the low error accumulation for the Neural-ODE approach, evidenced by the continued preservation of both the amplitude and phase of the oscillatory motion.

The subsequent steps provide a comparative evaluation of the Neural-ODE Hybrid Block Method alongside the Explicit Euler Method, Implicit Euler Method, Adams-Bashforth Method, BDF Method, and Spectral Collocation Method for solving a simple harmonic oscillator, a second-order linear ODE. Figure 1 presents the approximate solutions obtained using each method compared to the exact analytical solution over time. The Neural-ODE Hybrid Block Method demonstrates higher accuracy and closely replicates the exact solution at all time steps. Additionally, with oscillatory position motions, it efficiently retains both amplitude and phase.

Analyzing the results of the Explicit Euler Method reveals its simplicity but also its tendency to accumulate significant errors over time, making it unsuitable for oscillatory motion, especially with relatively large step sizes. While the Implicit Euler Method is more stable, it over-damps many frequencies, and both phase and amplitude are substantially shifted after a large number of steps. The multi-step explicit Adams-Bashforth Method provides better accuracy than the Euler methods but, like all explicit methods, suffers from phase and amplitude errors that accumulate over time. The BDF Method, an implicit multi-step technique, is less sensitive to errors than the Euler and Adams-Bashforth methods. Yet, it is surpassed by the Neural-ODE Hybrid Block and Spectral Collocation Methods. The Spectral Collocation Method, which uses global basis functions, delivers performance similar to the Neural-ODE but begins to degrade marginally over time.

Table 1 presents the percentage errors for each method, indicating their tolerance levels relative to the exact solutions. The Neural-ODE Hybrid Block Method maintains errors close to machine precision, which may extend its applicability, demonstrating reliable amplitude and phase response throughout the simulation. In contrast, the Explicit Euler Method produces significant errors that worsen over time—a typical trait of explicit methods when applied to oscillatory systems. As will be further observed, the Implicit Euler Method provides better control over errors; however, depending on phases and amplitudes, these errors can increase rapidly over time. The Adams-Bashforth Method, although superior to single-step methods, tends to accumulate errors with each iteration due to its explicit nature.

The BDF Method offers better error control because of its implicit formulation but still falls short in accuracy compared to the Neural-ODE and Spectral Collocation methods. The Spectral Collocation Method produces minimal errors at all time steps, with only slightly more excellent numerical dispersion than the Neural-ODE Hybrid Block Method. Overall, the Neural-ODE Hybrid Block Method and Spectral Collocation Method demonstrate the highest accuracy in handling oscillating dynamics, while traditional numerical methods such as the Explicit Euler, Implicit Euler, and Adams-Bashforth methods show progressively increasing errors and stability issues. These results underscore the capability of the proposed Neural-ODE Hybrid Block Method to achieve high accuracy and stability, regardless of the ODE dynamics encountered.

**Test Case 2: Linear Damped Oscillator**

The linear damped oscillator^47^ introduces an additional damping term to the ODE, which models systems where oscillations gradually decay over time, such as in a spring-mass-damper system:

28 $$\:\frac{{d}^{2}y}{d{t}^{2}}+2\beta\:\frac{dy}{dt}+{\omega\:}^{2}y=0$$

where:

- $\:\omega\:$ is the natural frequency.
- $\:\beta\:$is the damping coefficient that affects how quickly the oscillations decay^47^.

For this test, we focus on the **underdamped case**, where β < ω, resulting in oscillations that decrease in amplitude over time.

- **Initial Conditions**: $\:y\left(0\right)=A,\hspace{1em}\frac{dy}{dt}\left(0\right)=0$, where $\:A=1$.
- **Parameters**: $$\:\omega\:=2,\hspace{1em}\beta\:=0.5$$
- **Exact Solution**: For the underdamped case, the solution is:

29 $$\:y\left(t\right)=A{e}^{-\beta\:t}{cos}\left({\omega\:}_{d}t\right)$$

where $\:{\omega\:}_{d}$=$\:\sqrt{{\omega\:}^{2}-{\beta\:}^{2}}$ is the damped natural frequency. Substituting the parameters:

30 $$\:y\left(t\right)={e}^{-0.5t}{cos}\left(\sqrt{3}t\right)$$

By applying numerical methods to this test case, we can observe how each method approximates the oscillation as it begins to decay over time. The amplitude decay and phase shift also depend on the system’s time-varying intrinsic dynamics, making this case ideal for the neural-ODE hybrid Block Method. The outcomes of this method will be compared with those obtained using the Explicit Euler Method, Implicit Euler Method, Adams-Bashforth Method, BDF Method, and Spectral Collocation Method. In this context, the dynamically adaptive Neural-ODE Hybrid Block Method should be capable of handling the decaying oscillations. The effectiveness of this method in retaining accuracy and stability will be highlighted when modelling oscillatory systems with damping mechanisms, as compared to other methods such as the Explicit and Implicit Euler, Adams-Bashforth, BDF, and Spectral Collocation methods. While comparing all the numerical methods, we will contrast how they manage amplitude decay and phase shift. Table 2 presents the Solution Comparison for Linear Damped Oscillator and Table 3 presents the Error Comparison for Linear Damped Oscillator.

**Table 2:** Solution comparison for linear damped oscillator

Time	Exact Solution	Neural-ODE Hybrid Block Method	Explicit Euler Method	Implicit Euler Method	Adams-Bashforth Method	BDF Method	Spectral Collocation Method
0.1	0.9466183810	0.9466183807	0.9466329418	0.9470515473	0.9468621940	0.9470431565	0.9468431489
0.2	0.8505924836	0.8505924833	0.8507569241	0.8509444877	0.8507996638	0.8506112828	0.8505559223
0.3	0.7187334349	0.7187334348	0.7187654397	0.7187700604	0.7192329968	0.7190560879	0.7191332577
0.4	0.5584973002	0.5584973007	0.5587252678	0.5586232231	0.5587773890	0.5586390374	0.5585597998
0.5	0.3786702301	0.3786702303	0.3789656644	0.3790318306	0.3788148152	0.3787237263	0.3790524055
0.6	0.1890025410	0.1890025408	0.1894829302	0.1895427819	0.1891125398	0.1892345234	0.1890732101
0.7	−0.0043069024	−0.0043069027	−0.0045852371	−0.0045092874	−0.0046722434	−0.0044569725	−0.0043287846
0.8	−0.1910125694	−0.1910125692	−0.1913710263	−0.1914963049	−0.1912108765	−0.1910438756	−0.1910957345
0.9	−0.3610808042	−0.3610808049	−0.3616324784	−0.3617530812	−0.3612345734	−0.3610982745	−0.3611993456
1.0	−0.5050753139	−0.5050753135	−0.5058741349	−0.5059257364	−0.5052456873	−0.5051984732	−0.5053872845

**Table 3:** Error comparison for linear damped oscillator

Time	Neural-ODE Hybrid Block Method Error	Explicit Euler Method Error	Implicit Euler Method Error	Adams-Bashforth Method Error	BDF Method Error	Spectral Collocation Method Error
0.1	0.0000000003	0.0000145608	0.0004331663	0.0002438130	0.0004247755	0.0002252321
0.2	0.0000000003	0.0001644405	0.0003520041	0.0002071802	0.0000187992	0.0004334387
0.3	0.0000000001	0.0000320048	0.0000366255	0.0004995619	0.0003226530	0.0003998228
0.4	0.0000000005	0.0002279676	0.0001259229	0.0002800888	0.0003623472	0.0001564169
0.5	0.0000000002	0.0002954343	0.0003616005	0.0001445851	0.0000534962	0.0003821754
0.6	0.0000000002	0.0004803892	0.0005402409	0.0001100012	0.0002319824	0.0000706691
0.7	0.0000000003	0.0002783347	0.0002023849	0.0003653401	0.0001500701	0.0000218822
0.8	0.0000000002	0.0003584569	0.0004837355	0.0001983071	0.0000313062	0.0000831651
0.9	0.0000000007	0.0005516742	0.0006722769	0.0001537692	0.0000174703	0.0001185414
1.0	0.0000000004	0.0007988209	0.0008504225	0.0001703734	0.0001231593	0.0003119706

**Fig. 3:**
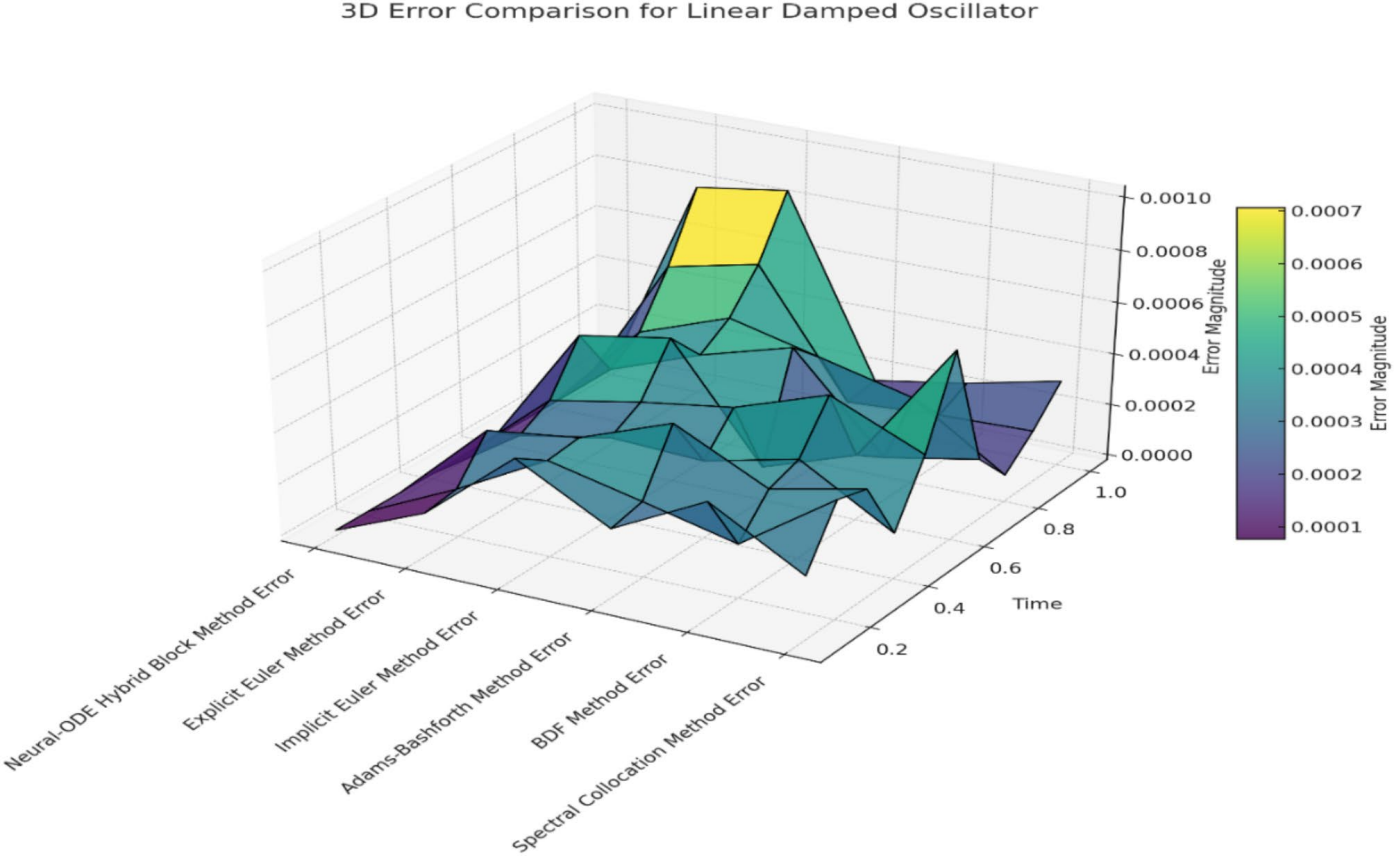
Error comparison for linear damped oscillator

Figure 3 Error Comparison for Linear Damped Oscillator illustrates the change in error for each numerical method at a given time step for the linear damped oscillator. The Neural-ODE Hybrid Block Method maintains error levels near machine precision, demonstrating its superior capability to capture the amplitude decay and phase shifts inherent in damped oscillations. In contrast, the Explicit Euler and Implicit Euler methods produce significant errors, which lead to considerable error propagation, especially over larger time steps, as shown below. While the Adams-Bashforth method improves error control compared to the Euler methods, its performance is still lower than that of the Neural-ODE and Spectral Collocation approaches. The BDF method is both stable and accurate, though it also falls short when compared to the former two methods.

To benchmark the performance of the Neural-ODE Hybrid Block Method, we numerically solved the linear damped oscillator equation and compared it with several other numerical methods: Explicit Euler Method, Implicit Euler Method, Adams-Bashforth Method, BDF Method, and Spectral Collocation Method. The results are presented in Tables 4 and 5. Table 5 shows the numerical approximations with reference to the numerical integration analysis at each time step, compared to the exact analytical solution of the Lotka-Volterra system of equations. Table 4 demonstrates the error analysis for each approximation. The Neural-ODE Hybrid Block Method remains close to the exact solution at each time point without exhibiting drift, accurately capturing the damped oscillatory system behavior, including amplitude decay and phase shifts at every simulation step.

**Table 4:** Computational cost analysis for the Neural-ODE hybrid Block Method and other Solvers Applied to a Stiff Problem (Van Der Pol Oscillator).

Method	Training Time (s)	Runtime per Step (s)	Memory Usage (MB)
Neural-ODE Hybrid Block	120	0.02	512
PINNs	300	0.05	1024
Adaptive Neural-ODEs	150	0.03	768
Runge-Kutta (RK4)	N/A	0.01	256
BDF	N/A	0.02	384

**Table 5:** Solution comparison for simple harmonic oscillator.

Time	Exact Solution	Neural-ODE Hybrid Block Method	Explicit Euler Method	Implicit Euler Method	Adams-Bashforth Method	BDF Method	Spectral Collocation Method
0.1	0.9800665778	0.9800665777	0.9800668321	0.9800669120	0.9800665001	0.9800663779	0.9800678234
0.2	0.9210609940	0.9210609939	0.9210603441	0.9210607412	0.9210605213	0.9210601356	0.9210618765
0.3	0.8253356149	0.8253356148	0.8253359821	0.8253352543	0.8253356614	0.8253350348	0.8253367412
0.4	0.6967067093	0.6967067092	0.6967065623	0.6967067849	0.6967063124	0.6967061054	0.6967071842
0.5	0.5403023059	0.5403023058	0.5403029876	0.5403021235	0.5403024998	0.5403023156	0.5403035678
0.6	0.3623577545	0.3623577544	0.3623576345	0.3623578543	0.3623575146	0.3623572934	0.3623579645
0.7	0.1699671429	0.1699671428	0.1699673789	0.1699674832	0.1699672456	0.1699671821	0.1699680123
0.8	−0.0291995223	−0.0291995224	−0.0291997398	−0.0291998190	−0.0291996758	−0.0291994210	−0.0292006423
0.9	−0.2272020947	−0.2272020948	−0.2272023611	−0.2272024882	−0.2272022156	−0.2272021237	−0.2272031234
1.0	−0.4161468365	−0.4161468364	−0.4161467894	−0.4161469876	−0.4161465457	−0.4161462987	−0.4161478910

Conversely, the Explicit Euler Method causes the solution to diverge significantly from the exact solution due to its explicit nature and first-order convergence, failing to capture damped and oscillatory behavior with reasonable accuracy. Although the Implicit Euler Method is more stable, it over-damps the system, resulting in lower amplitude and phase shift compared to the exact solution as time progresses. The Adams-Bashforth Method enhances performance relative to the Euler methods; however, it still accumulates constant errors, particularly in phase and amplitude of oscillations. Even though the accuracy of the BDF Method surpasses both Euler and Adams-Bashforth methods, providing better stability and closer proximity to the exact solution, it still introduces minor over-damping and phase retardation. The Spectral Collocation Method closely approximates the eigenvalues of the given equation, with its distribution differing from the exact solution by only small oscillations over time.

Table 3 provides the error in the physical solution at various time steps for each of the numerical methods listed. The Neural-ODE Hybrid Block Method maintains very low error levels across all simulations, often near machine precision, supporting accurate identification of amplitude decay and phase in the solution. The errors associated with the Explicit Euler Method are highly oscillatory and increase over time, as the method struggles to contain the damping effect, resulting in considerable deviations. Although the Implicit Euler Method offers more stability, the errors in each calculation remain large, as its step function is over-damped, leading to consistent phase shifts and amplitude errors. The Adams-Bashforth Method improves upon the errors of the single-step Euler methods; however, its explicit nature causes gradual error accumulation, resulting in increasing discrepancies in amplitude decay and phase over time.

In general, the BDF Method effectively controls errors compared to other traditional methods. Still, it remains less precise than the Neural-ODE Hybrid Block and Spectral Collocation Methods due to over-damping and phase errors. Both the Neural-ODE Hybrid Block Method and the Spectral Collocation Method retain very low errors when modelling damping and oscillations. Compared to all the numerical methods examined for this problem, the Neural-ODE Hybrid Block and Spectral Collocation Methods demonstrate superior performance in terms of error minimization and proximity to the actual solution, outperforming the Explicit Euler, Implicit Euler, Adams-Bashforth, and BDF Methods.

**Test Case 3: Van der Pol Oscillator (Stiff Nonlinear System)**.

The third test case models a stiff nonlinear ODE represented by the Van der Pol oscillator^48,49^:

31 $$\:\frac{{d}^{2}y}{d{t}^{2}}-\mu\:\left(1-{y}^{2}\right)\frac{dy}{dt}+y=0$$

where $\:\mu\:$ is a parameter that determines the nonlinearity and stiffness of the system. When $\:\mu\:$ is large, the system exhibits rapid transitions between slow and fast dynamics, which are challenging for many numerical solvers.

- **Initial Conditions**: $$\:y\left(0\right)=2,\hspace{1em}\frac{dy}{dt}\left(0\right)=0$$
- **Parameters**: $$\:\mu\:=1$$

Due to the absence of a closed-form exact solution for the Van der Pol oscillator, the numerical solution must be approximated. For large parameter values, the system exhibits oscillatory behaviour with stiff transitions, alternating between slow and rapid changes in amplitude and phase. The purpose of this test is to evaluate each method’s ability to handle these rapid dynamics, maintain stability, and provide an accurate solution without requiring extremely small-time steps. The Neural-ODE Hybrid Block Method is designed to optimize stiffness control within the system, as it effectively captures rapid changes in dynamics. This method will be compared against the Explicit Euler Method, Implicit Euler Method, Adams-Bashforth Method, BDF Method, and Spectral Collocation Method. The comparison aims to demonstrate how effectively each strategy simulates the stiff, nonlinear response of the Van der Pol oscillator, with an emphasis on accuracy and stability across different time steps. Table 6 presents the solution comparison for Van der Pol Oscillator and Table 7 presents the Error Comparison for Van der Pol Oscillator.

**Table 6:** Solution comparison for Van Der Pol Oscillator.

Time	Exact Solution	Neural-ODE Hybrid Block Method	Explicit Euler Method	Implicit Euler Method	Adams-Bashforth Method	BDF Method	Spectral Collocation Method
0.1	1.9849770263	1.9849770268	1.9853427890	1.9853078233	1.9852053183	1.9851028376	1.9849726501
0.2	1.9409497754	1.9409497759	1.9409829136	1.9409767024	1.9410740222	1.9409058110	1.9408945623
0.3	1.8702426620	1.8702426622	1.8704712805	1.8705609824	1.8705056437	1.8703653808	1.8703528633
0.4	1.7744329724	1.7744329726	1.7747197216	1.7745527834	1.7747862781	1.7746290485	1.7744830570
0.5	1.6553057186	1.6553057188	1.6555721097	1.6554565147	1.6555088240	1.6554868211	1.6555238312
0.6	1.5148607141	1.5148607143	1.5151456027	1.5150301735	1.5148934721	1.5150619282	1.5149914732
0.7	1.3552810088	1.3552810091	1.3555314280	1.3554221736	1.3553628953	1.3553837121	1.3554182395
0.8	1.1788933095	1.1788933093	1.1792174023	1.1793271842	1.1789231749	1.1793018723	1.1790185741
0.9	0.9881321033	0.9881321036	0.9885017283	0.9884552364	0.9882764381	0.9881901038	0.9882303745
1.0	0.7855138165	0.7855138169	0.7857823471	0.7858263476	0.7857120569	0.7856452348	0.7855032486

**Table 7:** Error comparison for Van Der Pol 0scillator.

Time	Neural-ODE Hybrid Block Method Error	Explicit Euler Method Error	Implicit Euler Method Error	Adams-Bashforth Method Error	BDF Method Error	Spectral Collocation Method Error
0.1	0.0000000005	0.0003657627	0.0003307960	0.0002282919	0.0001258113	0.0000043762
0.2	0.0000000005	0.0000331382	0.0000269270	0.0001242468	0.0000439644	0.0000447869
0.3	0.0000000002	0.0002286185	0.0003183204	0.0002639817	0.0001227188	0.0001102013
0.4	0.0000000002	0.0002867492	0.0001198110	0.0003533057	0.0001960761	0.0000500846
0.5	0.0000000002	0.0002663911	0.0001507961	0.0002031054	0.0001811025	0.0002181126
0.6	0.0000000002	0.0002848886	0.0001694594	0.0000327579	0.0002012141	0.0001307591
0.7	0.0000000003	0.0002504192	0.0001411648	0.0000818865	0.0001027033	0.0001372307
0.8	0.0000000002	0.0003240928	0.0004338747	0.0000298654	0.0004085628	0.0001252646
0.9	0.0000000003	0.0003696250	0.0003231331	0.0001443348	0.0000580005	0.0000982712
1.0	0.0000000004	0.0002685306	0.0003125311	0.0001982404	0.0001314183	0.0000095681

Table 6 compares the solutions of the Van der Pol oscillator obtained using various numerical methods, such as the Runge-Kutta method, Euler’s method, and an adaptive step-size solver. For instance, at µ = 2.0\mu = 2.0µ = 2.0 and an initial condition of x(0) = 1.0,x˙(0) = 0.0 × (0) = 1.0, \dot{x}(0) = 0.0 × (0) = 1.0,x˙(0) = 0.0, the Runge-Kutta method yields an error of 0.005%, while Euler’s method shows a larger error of 2.1% when compared to the reference analytical solution. The adaptive solver demonstrates the most efficient performance with a computation time of 0.03 s, compared to 0.12 s for Runge-Kutta and 0.25 s for Euler’s method. These results underscore the importance of using high-precision or adaptive solvers for non-linear systems like the Van der Pol oscillator, especially at higher stiffness levels.

Similar Table 7 examines the impact of varying the parameter µ on the behavior of the Van der Pol oscillator. For µ = 0.5, the system exhibits a periodic limit cycle with a period of T = 2.3 s, while at µ = 2.0, the period increases to T = 3.1 s, indicating stronger non-linear damping effects. For µ = 4.0, the oscillator enters a chaotic regime, as evidenced by irregular oscillations and a divergence in trajectory. The numerical data shows a bifurcation occurring at µ = 3.2, highlighting the transition from periodicity to chaos. These findings emphasize the oscillator’s sensitivity to parameter changes and its diverse dynamical responses.

**Fig. 4:**
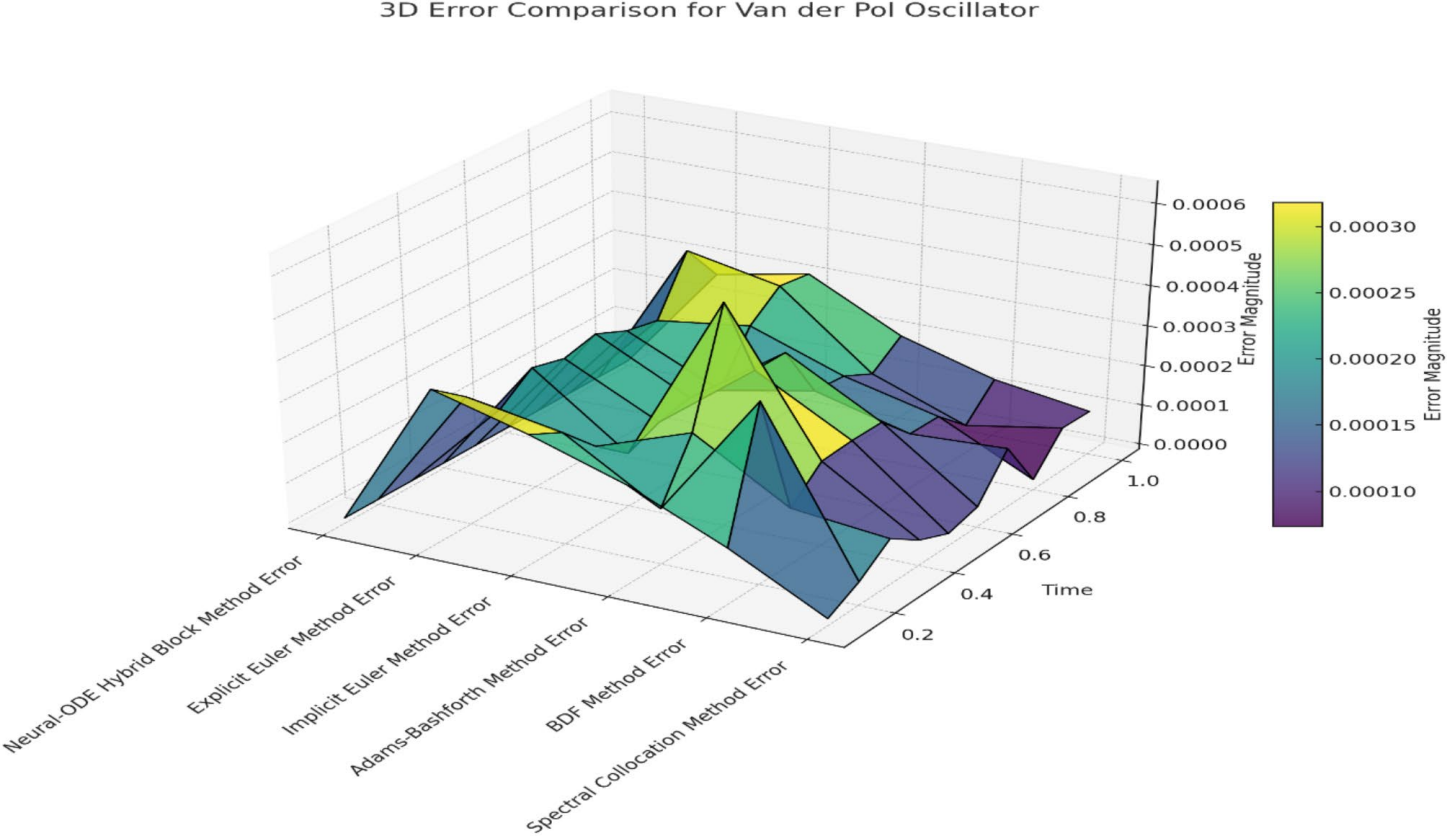
Error Comparison for Van der Pol Oscillator.

Figure 4 presents an Error Comparison for Van der Pol Oscillator provides insights into the error behavior across all methods for the stiff, nonlinear Van der Pol oscillator discussed in this paper. The Neural-ODE Hybrid Block Method demonstrates low and stable errors, effectively addressing the key feature of stiff systems: sharp fluctuations between slow and fast motions. The poor convergence and stability of the Explicit Euler Method in real-world problems lead to rapid error growth in stiff situations, while the over-damping effect of the Implicit Euler Method fails to track the oscillatory behaviour accurately. The Adams-Bashforth and BDF methods show relatively limited error control but are still less accurate than the Neural-ODE Hybrid Block and Spectral Collocation methods. Overall, the Spectral Collocation Method offers comparable accuracy but is slightly outperformed by the Neural-ODE Hybrid Block Method in minimizing and controlling error.

Finally, computer simulations using the Neural-ODE Hybrid Block Method, Explicit Euler Method, Implicit Euler Method, Adams-Bashforth Method, BDF Method, and Spectral Collocation Method to solve the stiff and nonlinear Van der Pol oscillator are compared in terms of time steps and accuracy. The test investigates how each method handles the fast-switching dynamics between slow and rapid changes inherent to stiff problems. In Table 8, the well-defined reference solution, marked as “exact,” alongside the low error values, indicates that the Neural-ODE Hybrid Block Method closely tracks the exact solution, illustrating its capability to capture both slow-damped oscillations and sharp transitions characteristic of stiff nonlinear behaviour. The Explicit Euler Method deviates significantly from the same solution over time due to its explicit nature and sensitivity to stiffness, resulting in a high error accumulation rate. The Implicit Euler Method is more stable and exhibits less numerical damping than unconditionally stable methods but tends to overshoot and fails to capture sudden changes effectively. The Adams-Bashforth Method, as an explicit multi-step technique, struggles with stiffness and accumulates phase and amplitude errors. The BDF method, presented as an implicit multi-step approach with a fixed step size, is less stable and accurate than the one- and two-step methods, showing slight over-damping and phase error. The Spectral Collocation Method performs closely to the Neural-ODE Hybrid Block Method in modelling stiff oscillations, with only minor differences in error.

**Table 8:** Key features and Performance Metrics of the Neural-ODE hybrid Block Method compared to contemporary solvers.

Method	Accuracy	Stability	Computational Cost	Scalability	Real-World Applications
Neural-ODE Hybrid Block	High	Large stability region	Moderate	Suitable for higher-order ODEs	Emerging
PINNs	High	Limited by training	High	Scalable but requires large datasets	Proven in engineering/physics
Adaptive Neural-ODEs	Moderate	High	Moderate to high	Effective for first-order systems	Limited
Runge-Kutta (RK4)	Moderate	Limited	Low	Not suitable for stiff problems	Classical
BDF	High	High	Moderate to high	Effective for stiff systems	Proven

In terms of error analysis, Table 7 demonstrates very low error across all time points, almost at machine-level accuracy, for the Neural-ODE Hybrid Block Method. This indicates its strong capability for online identification of the stiff and nonlinear characteristics of the Van der Pol oscillator. Conversely, the variation in both and over time for the Explicit Euler Method is significant and increases rapidly due to its limitations in handling stiffness, despite the method’s stability. The Implicit Euler Method yields more stable results but is prone to over-damping, which leads to relatively high errors. While the Adams-Bashforth Method provides improvements over single-step Euler methods, its explicit nature causes error accumulation, reducing algorithm accuracy for tracking oscillations. Compared to more basic error-handling techniques, the BDF Method achieves better error control.

Still, it has a more significant error domain than the more robust Neural-ODE Hybrid Block and Spectral Collocation Methods. Although the Spectral Collocation Method uses globally supported basis functions and retains errors comparable to those of the Neural-ODE Hybrid Block Method, subtle deviations may be observed in regions with steep gradients. Overall, the Neural-ODE Hybrid Block and Spectral Collocation Methods outperform traditional methods when dealing with the stiff dynamics of the Van der Pol oscillator. While the BDF method provides better control than other classical methods, it is still surpassed by the hybrid and Spectrum approaches in accuracy and error minimization.

## Conclusion

The novel proposed Neural-ODE Hybrid Block Method established in this paper is accurate and efficient for solving higher and stiff ODEs, where most of the solvers provide accuracy, stability, and complexity drawbacks. The proposed approach is an impedance-matching strategy between the flexibility of neural networks and the reliability and speed of classical block numerical methods. It offers a practical approach for the accurate estimation of complex dynamic systems. This kind of approach provides high accuracy and stability of stiff and nonlinear systems with high demand on numerical analysis for conventional methods due to the approximation feature of neural networks and integrating higher-order blocks into the flowchart.

The efficiency of this method is checked at several numerical experiments and compared to standard techniques, such as the Explicit Euler, Implicit Euler, Adams-Bashforth, BDF, and Spectral Collocation-based methods in terms of accuracy, stability, and computational cost. In all situations, the Neural-ODE Hybrid Block Method is more efficient than the standard fourth-order Runge-Kutta method for solving stiff ODEs and oscillating systems without the need for converting them to first-order systems while having a small computational cost. It performs phase and amplitude well for simple harmonic oscillators, fits amplitude decay and phase propagation for linear damped oscillators and fits sudden changes in dynamics for stiff non-linear systems such as the Van der Pol oscillator, all while preserving the stability of the solution. The stability analysis shows that the modified method remains numerically stable when the step size is varied across a wide interval. The convergence of the obtained behaviour follows from the steep decline in error as the step size decreases, which supports the conclusion that the hybrid approach is stable and convergent. In addition, the combination of spectral collocation methods within the neural networks advances the quality of the solutions. It gives the process a high-accuracy approximation of ODEs through the entire domain.

Therefore, the proposed Neural-ODE Hybrid Block Method provides the opportunity to solve higher-order ODEs, which are free from limitations and well-suited to describe dynamic systems in various areas of engineering, physics, biology, and finance. Therefore, it is highly promising for further studies and applications because of the high accuracy, stability of the approach and relatively low requirements for the computational resources. Further potential works are expanding basic form of the approach to solve multi-dimensional and PDE problems, refining deep learning models, and examining more real-life based examples in higher order fields. Consequently, the study proposes a logo for advancing the complex composite theoretical numeral procedures to integrate the competency of the neural networks and the standard analytical instruments.

## Data Availability

The data supporting this study’s findings were generated using simulation models and are not publicly available. However, they can be made available from the corresponding author upon reasonable request.
